# Differential effects of voluntary exercise and Totum-448, a plant-based formulation, in a hamster model of MASLD

**DOI:** 10.1038/s41598-026-43177-5

**Published:** 2026-03-09

**Authors:** Vivien Chavanelle, Gaël Ennequin, Doriane Ripoche, Yolanda F. Otero, Florian Le Joubioux, Thierry Maugard, Valérie Hervieu, Sébastien Peltier, Pascal Sirvent

**Affiliations:** 1 Valbiotis R&D Center, 4 rue Eric Tabarly Périgny, Valbiotis, France; 2 Laboratoire des Adaptations Métaboliques à l’Exercice en conditions Physiologiques et Pathologiques (AME2P), Université Clermont Auvergne, UR 3533, Clermont-Ferrand, 63000 France; 3 Equipe BCBS (Biotechnologies et Chimie des Bioressources pour la Santé), La Rochelle, Université, UMR CNRS 7266 LIENSs, La Rochelle, France; 4 Department of Pathology, Hospices Civils de Lyon, Lyon, Auvergne-Rhône-Alpes France; 5 Université de Lyon, Lyon, Auvergne-Rhône-Alpes France

**Keywords:** MASLD, Voluntary exercise, Totum-448, Polyphenols, Diseases, Health care, Medical research, Physiology

## Abstract

Lifestyle interventions constitute first-line strategies for the management of metabolic dysfunction-associated steatotic liver disease (MASLD), with dietary modification and increased physical activity forming the core evidence-based approaches. In this framework, plant-based supplements are being investigated as potential adjunctive strategies. Totum-448, a polyphenol-rich plant formulation, has shown promising effects in preclinical studies. Here, we evaluated the effects of Totum-448, voluntary exercise (Vex), and their combination in a hamster model of MASLD. Fifty-four male golden Syrian hamsters were fed either a normal diet (ND) or a Western diet (WD) for six weeks. WD-fed animals were then randomized to receive WD alone, WD supplemented with Totum-448 (5% w/w), WD with Vex, or WD with both interventions for an additional five weeks. We hypothesized that only the combined intervention would elicit measurable benefits. Contrary to this hypothesis, distinct effects were observed for each intervention: Totum-448 reduced circulating lipid levels and downregulated hepatic markers of inflammation and fibrosis, whereas Vex improved body composition, increased energy expenditure, and reduced hepatic lipid content. No significant improvements were detected in histological markers of inflammation or fibrosis. The combined intervention did not provide additional benefits beyond those of the individual interventions. In conclusion, Totum-448 and Vex exert complementary effects in MASLD. Although no additive effects were observed, the combined intervention provided the broadest overall protection by preserving the benefits of each intervention alone. These findings support the relevance of combining exercise with plant-based nutritional strategies in the management of MASLD.

**Supplementary Information:**

The online version contains supplementary material available at 10.1038/s41598-026-43177-5.

## Introduction

Metabolic dysfunction-associated steatotic liver disease (MASLD), formerly known as non-alcoholic fatty liver disease (NAFLD)^1^, is now recognized as a major cause of chronic liver injury, largely driven by the rising prevalence of obesity and metabolic syndrome^2,3^. Its global prevalence is estimated at 30–32% and is expected to increase further as the obesity pandemic continues to expand^4,5^. MASLD encompasses a spectrum from simple steatosis to more severe conditions marked by inflammation, hepatocyte ballooning, and fibrosis, which can ultimately progress to cirrhosis and liver failure^6^. Despite its clinical importance, treatment options are limited; to date, only one pharmacological agent (Resmetirom, a thyroid hormone receptor β agonist) has received approval from the U.S. Food and Drug Administration^7^. Consequently, lifestyle modifications, particularly dietary adjustments and physical activity, remain the cornerstone of MASLD management. In this framework, Totum-448, a polyphenol-rich supplement composed of extracts from olive leaf (*Olea europaea*), bilberry (*Vaccinium myrtillus*), artichoke leaf (*Cynara scolymus*), chrysanthellum (*Chrysanthellum indicum subsp. afroamericanum B.L. Turner*), black pepper (*Piper nigrum*), and choline, was developed to prevent the MASLD progression. Totum-448 has shown promising results on hepatic steatosis and inflammation in both hamster^8^ and mouse^9^ models of the disease. Lifestyle modifications remain the cornerstone of MASLD management, with physical activity holding a central role. Current guidelines from the European Association for the Study of the Liver (EASL) and other health authorities recommend 150–200 min of moderate-intensity exercise per week for individuals with or at risk of MASLD^10–12^, ideally combined with dietary interventions to achieve weight loss reduction^13^. The individual variability in response to exercise^14^ and poor long-term adherence^15^ prompt the need for complementary strategies to enhance effectiveness and sustainability.

In this context, we sought to evaluate the combined effects of Totum-448 and chronic exercise in a diet-induced MASLD hamster model. We used the previously established effective dose of Totum-448 (5% incorporated into the diet^8^, and implemented voluntary exercise (Vex) using a freely accessible running wheel^16–18^. Our aim was to assess the effects of both interventions, alone or in combination, over a relatively short period of 5 weeks, following a 6-week induction of MASLD. This contrasts with our prior study, in which beneficial effects of Totum-448 were observed after 12 weeks of supplementation. Given the more aggressive study design and limited intervention period, we hypothesized that only the combined treatment would produce measurable improvements in MASLD, whereas individual interventions might be insufficient under these conditions.

## Results

### MASLD induction

**Fig. 1:**
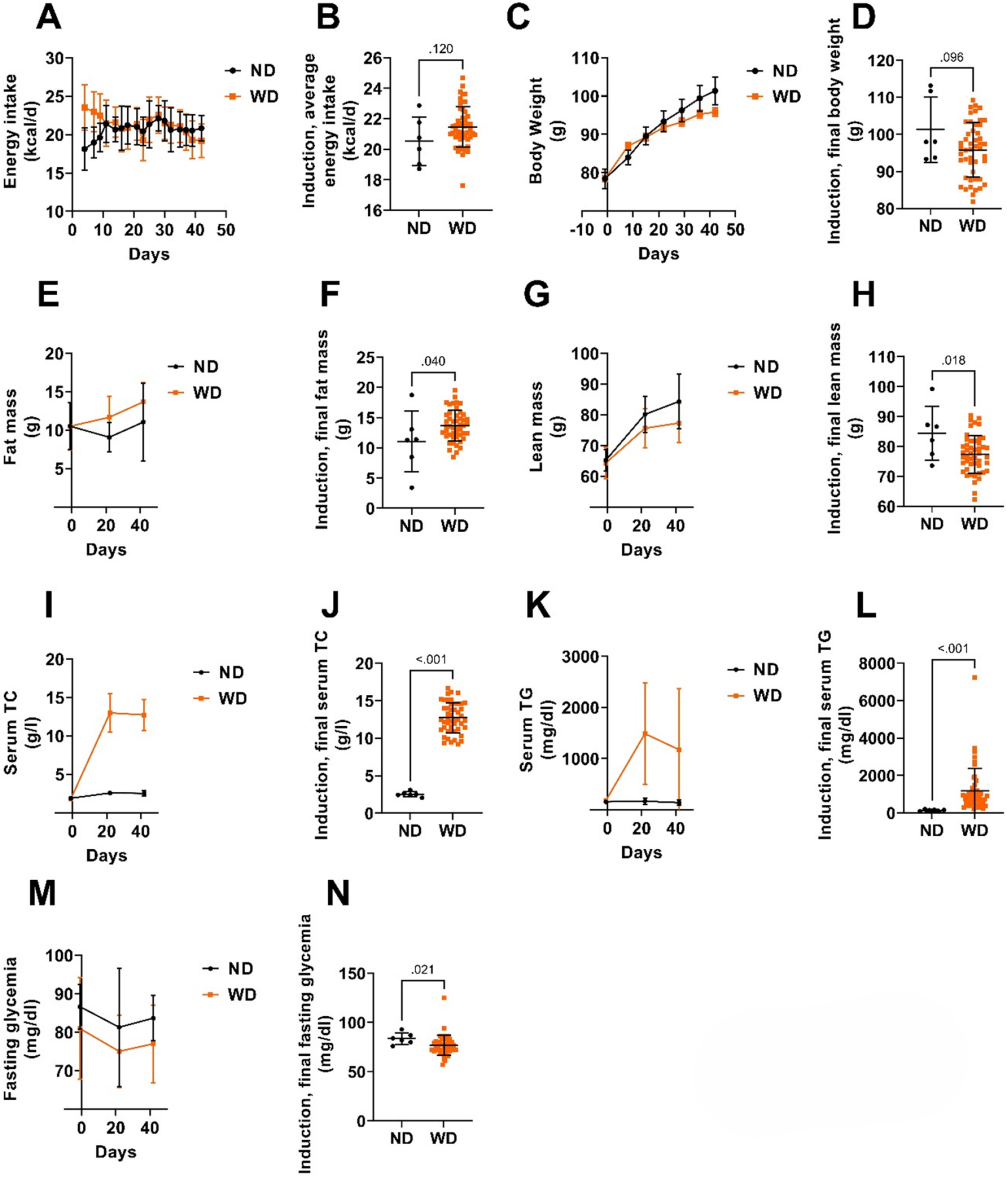
Induction of MASLD, from day 1 to day 42. (**A**) Daily energy intake over induction period. (**B**) Average daily energy intake. (**C**) Body weight over induction period. (**D**) Final body weight at the end of the induction period (day 42). (**E**) Fat mass over induction period. (**F**) Final fat mass at the end of the induction period (day 42). (**G**) Lean mass over induction period. (**H**) Final lean mass at the end of the induction period (day 42). (**I**) Serum TC over induction period. (**J**) Final serum TC at the end of the induction period (day 42). (**K**) Serum TG over induction period. (**L**) Final serum TG at the end of the induction period (day 42). (**M**) Fasting glycemia over induction period. (**N**) Final fasting glycemia at the end of the induction period (day 42). *N* = 6–48 animals. For the sake of clarity and concision, statistical analyses were performed on average or final timepoint only. Student’s t-test or Welch-corrected Student’s t-test or Mann-Whitney test. ND: normal diet. TC: total cholesterol. TG: triglycerides. WD: western diet.

MASLD was induced in 48 hamsters through 42 days of Western diet (WD) feeding. Energy intake over time is shown in Fig. 1A, and average intake during the induction period did not differ significantly between groups (Fig. 1B). Body weight trajectories are presented in Fig. 1C. At the end of the induction period, body weight in the WD group was slightly lower than in the ND group, although this difference was not statistically significant (95.8 ± 7.4 vs. 101.3 *±* 8.8 g, *p* = 0.096, Fig. 1D). In contrast, WD feeding significantly increased fat mass (13.7 ± 2.5 vs. 11.1 ± 5.1 g, *p* = 0.040, Fig. 1F) and reduced lean mass (77.4 ± 6.4 vs. 84.4 ± 9.0 g, *p* = 0.018, Fig. 1H). Serum total cholesterol and triglycerides were also significantly elevated in WD-fed hamsters (*p* < 0.001, Fig. 1J and L). Fasting glycemia, however, was lower in the WD group compared to ND (*p* = 0.021, Fig. 1N), although this difference may have existed at baseline (Fig. 1M). In summary, 42 days of WD induced adverse changes in body composition and circulating lipids, despite similar energy intake and body weight, while fasting glycemia was not negatively affected.

### Effects of interventions post-induction

After 42 days of MASLD induction in group WD, animals were randomized into 4 intervention groups (WD, WD-T448, WD-Vex or WD-Vex-T448) for 36 ± 1 additional days (depending on the day of euthanasia), while the 6 animals in group ND carried on with the same diet.

### Energy intake, body weight and composition

**Fig. 2:**
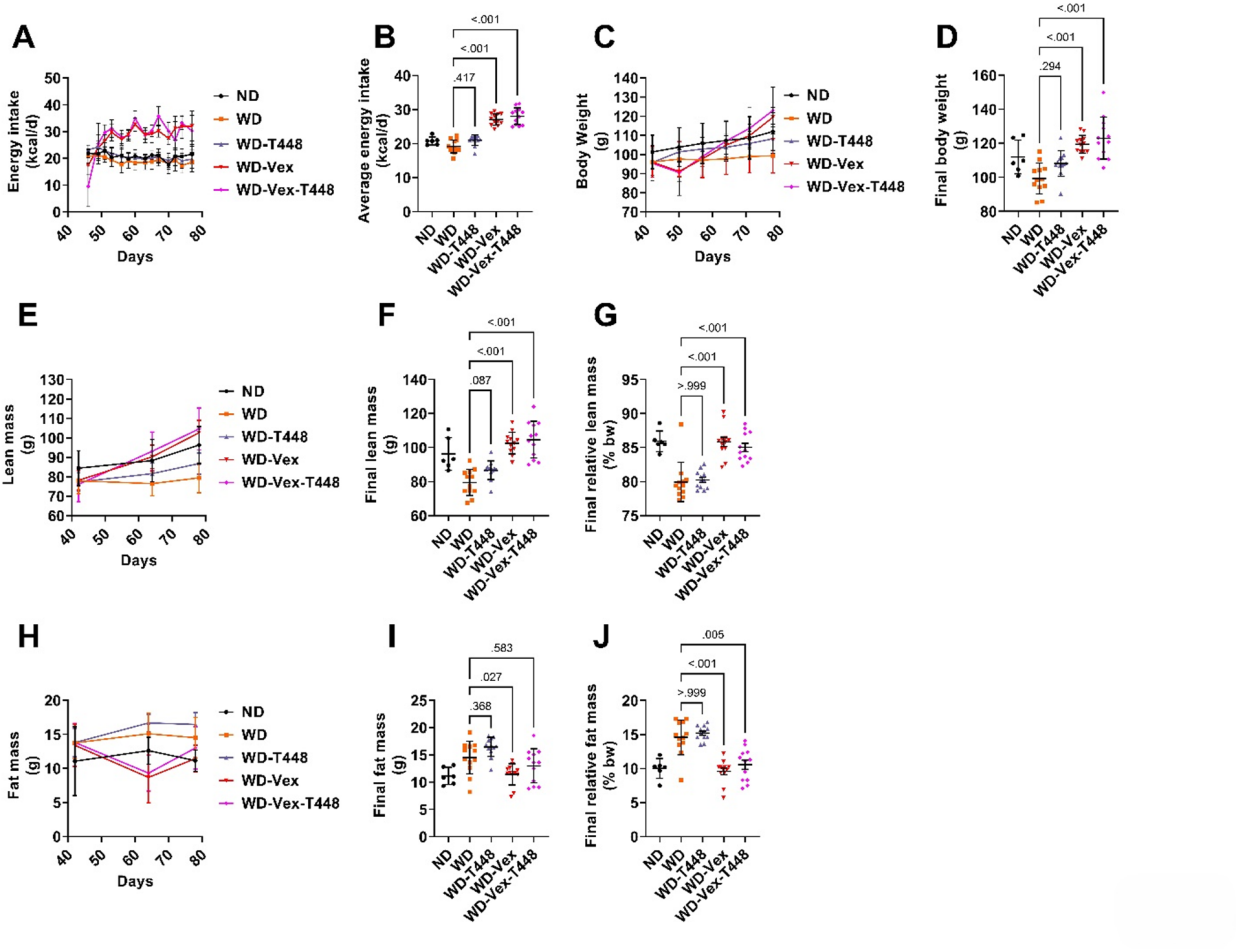
Energy intake, body weight, and body composition following interventions (from day 43 until the end of the study (day 78 ± 1). (**A**) Daily energy intake over intervention period. (**B**) Average energy intake over intervention period. (**C**) Body weight over intervention period. (**D**) Final body weight at the end of the intervention period. (**E**) Lean mass over intervention period. (**F**) Final lean mass at the end of the intervention period. (**G**) Relative final lean mass (body-weight normalized). (**H**) Fat mass over intervention period. (**I**) Final fat mass at the end of the intervention period. (**J**) Relative final fat mass (body-weight normalized). *N* = 6–12 animals. For the sake of clarity and concision, statistical analyses were performed on average or final timepoint only. Pair-wise comparisons (Šidák’s, Dunnett’s, or Dunn’s post-hoc tests) are indicated only if one-way ANOVA, Welch-corrected one-way ANOVA, or Kruskal-Wallis test was significant, respectively (*p* < 0.05). No pairwise comparison with group ND was carried out, this group is shown for reference only. ND: normal diet. WD: western diet. WD-T448: western diet + Totum-448. WD-Vex: western diet + voluntary exercise. WD-Vex-T448: western diet + voluntary exercise + Totum-448.

Daily energy over study period is presented in Fig. 2A. As observed during the induction period, group WD did not display any increase in energy intake. However, average daily energy intake was significantly higher in exercising groups (WD-Vex and WD-Vex-T448) compared to WD (27.1 ± 1.7 and 28.1 ± 2.5 vs. 19.2 ± 1.9 kcal/d, *p* < 0.001, Fig. 2B). Totum-448 supplementation alone did not affect energy intake (WD-T448 vs. WD, *p* = 0.417, Fig. 2B). Body weight trajectories are presented in Fig. 2C. Final body weight was significantly increased in WD-Vex and WD-Vex-T448 compared to WD (119.4 ± 5.2 and 123.0 ± 12.3 vs. 99.4 ± 9.0 g, *p* < 0.001 and *p* < 0.001, Cohen’s d: 2.73 and 2.19, respectively, Fig. 2D) with no effect observed for Totum-448 alone. In line with this, lean mass was increased in both exercising groups (WD-Vex and WD-Vex-T448) vs. WD, at the end of the study (102.6 ± 6.4 and 104.6 ± 10.8 vs. 79.4 ± 7.7 g, *p* < 0.001 and *p* < 0.001, Cohen’s d: 3.29 and 2.69, respectively) while WD-T448 showed only a non-significant increase (86.8 ± 5.5 vs. 79.4 ± 7.7 g, *p* = 0.087, Cohen’s d: 0.79, Fig. 2F). When normalized by body weight, relative lean mass was also found increased in both exercising groups (WD-Vex and WD-Vex-T448) vs. WD, at the end of the study (85.84 ± 2.33 and 85.02 ± 2.11 vs. 79.94 ± 2.89%, *p* < 0.001 and *p* < 0.001, Cohen’s d: 2.25 and 2.01, respectively, Fig. 2G), with no effect of Totum-448. Fat mass was significantly reduced in WD-Vex compared to WD (11.4 ± 2.0 vs. 14.5 ± 3.0 g, *p* = 0.027, Cohen’s d: -1.22, Fig. 2I). With body weight normalization, relative fat mass was significantly decreased in both exercising groups (WD-Vex and WD-Vex-T448) vs. WD, at the end of the study (9.60 ± 1.73 and 10.58 ± 2.37 vs. 14.58 ± 2.55%, *p* < 0.001 and *p* = 0.005, Cohen’s d: -2.29 and − 1.63, respectively, Fig. 2J).

### Muscle and fat pad mass

**Fig. 3:**
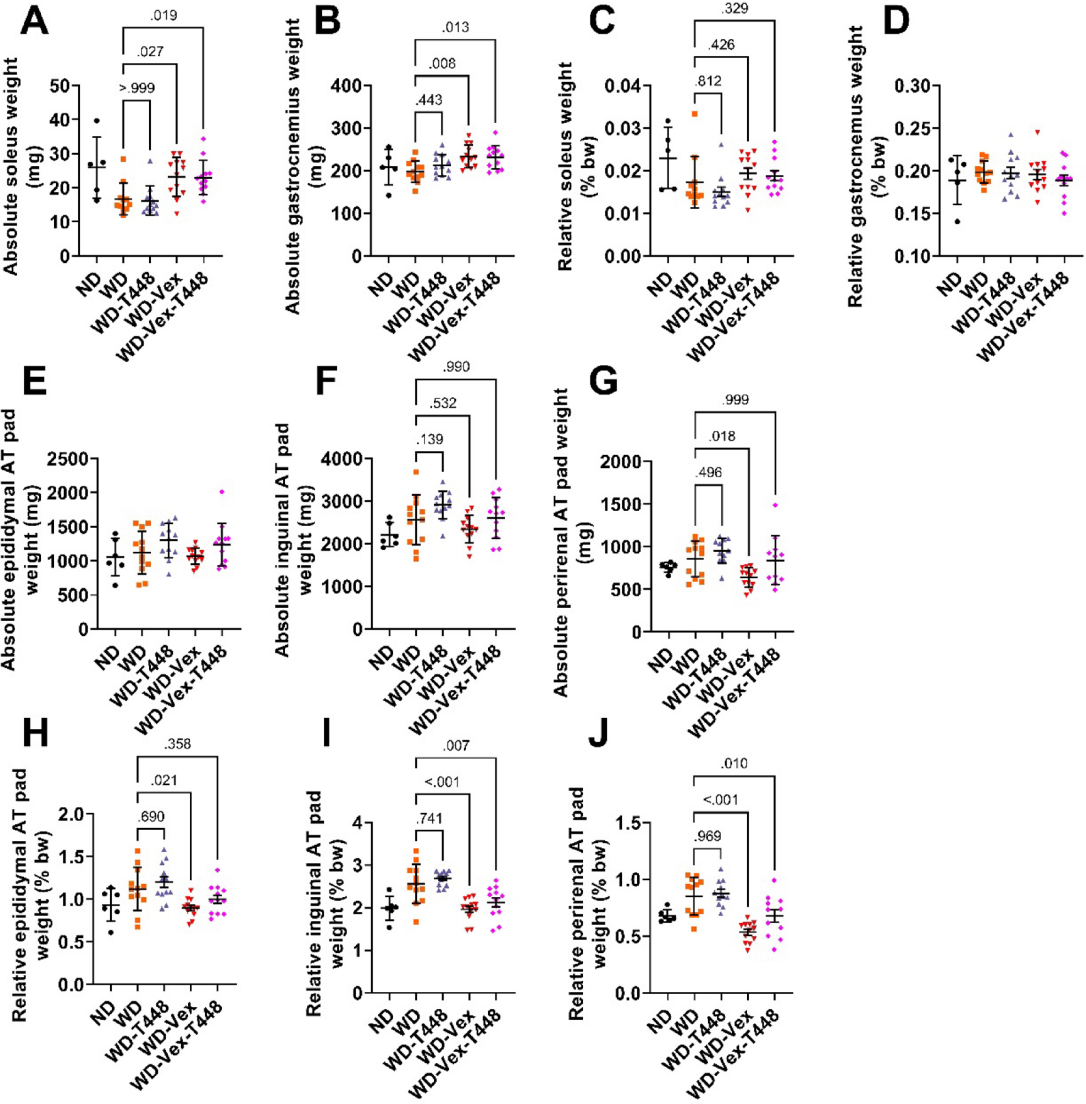
Absolute and relative weight of muscles and adipose tissue pads. (**A**) Absolute soleus muscle weight. (**B**) Absolute gastrocnemius muscle weight. (**C**) Relative soleus muscle weight (body weight normalized). (**D**) Relative gastrocnemius muscle weight (body weight-normalized). (**E**) Absolute weight of epididymal AT pads. (**F**) Absolute weight of inguinal AT pads. (**G**) Absolute weight of perirenal AT pads. (**H**) Relative weight of epididymal AT pads (body weight-normalized). (**I**) Relative weight of inguinal AT pads (body weight-normalized). J: Relative weight of perirenal AT pads (body weight-normalized). *N* = 6–12 animals. Pair-wise comparisons (Šidák’s, Dunnett’s, or Dunn’s post-hoc tests) are indicated only if one-way ANOVA, Welch-corrected one-way ANOVA, or Kruskal-Wallis test was significant, respectively (*p* < 0.05). No pairwise comparison with group ND was carried out, this group is shown for reference only. AT: adipose tissue. ND: normal diet. WD: western diet. WD-T448: western diet + Totum-448. WD-Vex: western diet + voluntary exercise. WD-Vex-T448: western diet + voluntary exercise + Totum-448.

Consistent with lean mass changes, absolute soleus and gastrocnemius muscle mass were significantly higher in both exercising groups (WD-Vex and WD-Vex-T448) compared to WD (soleus: 23.1 ± 5.7 and 23.0 ± 5.0 vs. 16.7 ± 4.6 mg, *p* = 0.027 and 0.019; gastrocnemius: 233.3 ± 26.6 and 231.2 ± 27.7 vs. 197.3 ± 24.5 mg, *p* = 0.018 and 0.013, Fig. 3A-B). These significant differences disappeared with body weight normalization (Fig. 3C-D), suggesting that these changes primarily reflect the overall increase in body weight driven by lean mass. In contrast, absolute epididymal and inguinal fat pad weights were not significantly different among groups (Fig. 3E-F), but perirenal fat pad weight was significantly reduced in WD-Vex compared to WD (638.7 ± 113.6 vs. 853.8 ± 209.0 mg, *p* = 0.018, Fig. 2M). Normalization by body weight indicated a significant decrease in relative epididymal fat mass pad in WD-Vex, compared to WD (0.89 ± 0.11 vs. 1.12 ± 0.25%, *p* = 0.021, Fig. 3H), a reduction of relative inguinal fat pad weight in WD-Vex and WD-Vex-T-448 vs. WD (1.96 ± 0.26 and 2.12 ± 0.37 vs. 2.68 ± 0.17%, *p* < 0.001 and *p* = 0.007, Fig. 3I), and a diminution of relative perirenal fat pad weight in WD-Vex and WD-Vex-T448, compared with WD (0.53 ± 0.09 and 0.68 ± 0.18 vs. 0.88 ± 0.11%, *p* < 0.001 and *p* = 0.010, Fig. 3J). Taken together, these results indicate that voluntary exercise primarily induced a global shift in body composition rather than selective changes in individual tissues. The loss of significance in muscle mass after normalization suggests that muscle accretion scaled proportionally with overall body weight, driven by increased lean mass. In contrast, body-weight–normalized adipose tissue weights revealed reduced relative adiposity in exercising animals, despite unchanged absolute values. No depot-specific effects were observed, supporting a generalized effect of exercise on body composition. Totum-448 alone elicited no significant effect.

### Vex quantification and Totum-448 intake

**Fig. 4:**
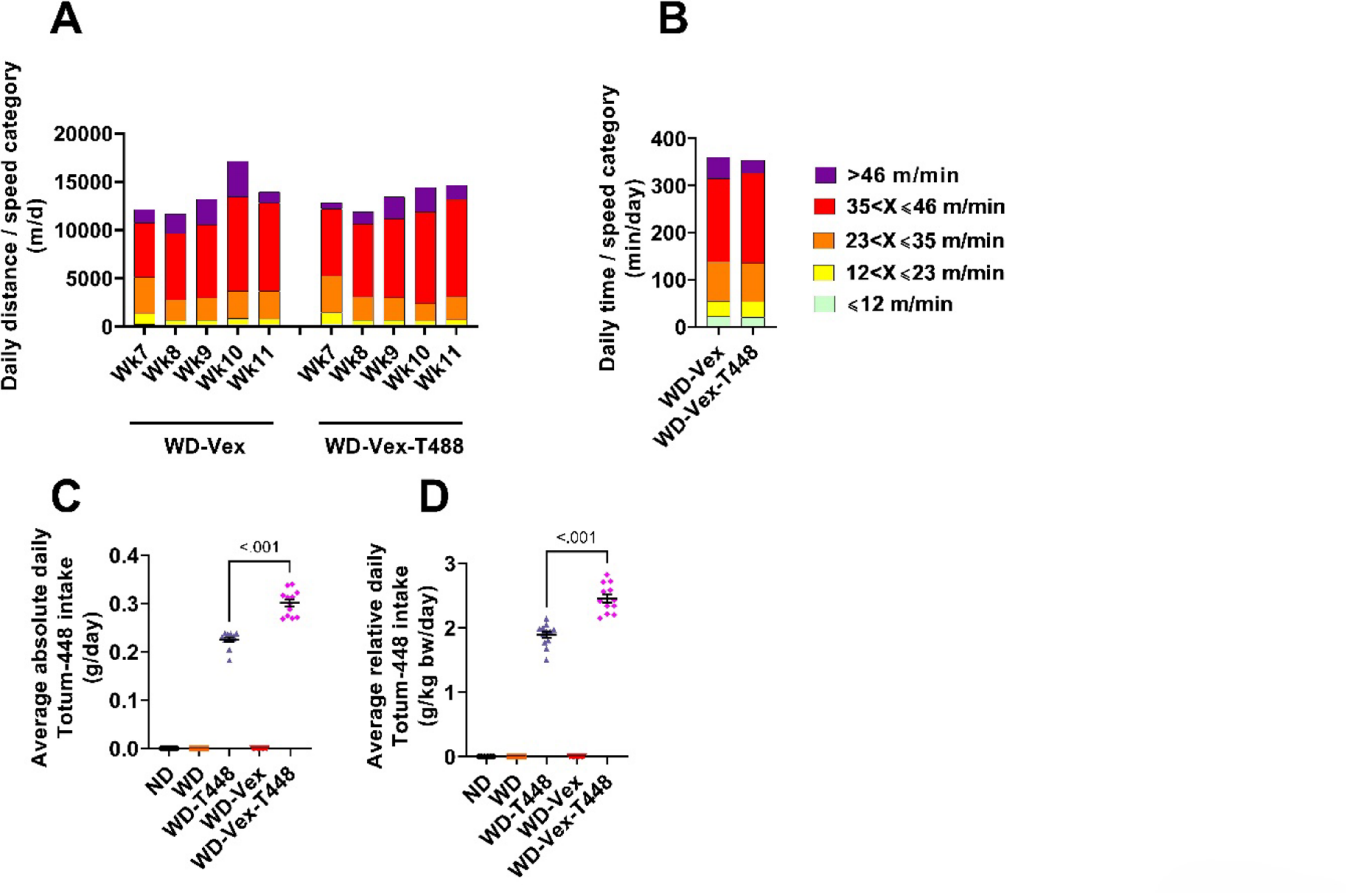
Vex monitoring in the activity wheels and Totum-448 intake. (**A**) Speed was classified into 5 categories: ⩽12 m/min, 12 < X⩽23 m/min, 23 < X⩽35 m/min, 35 < X⩽46 m/min, or > 46 m/min, and average daily distance per week is presented as a function of these speed categories. (**B**) Average daily time spent running in the different speed categories, overall. (**C**) Average absolute daily Totum-448 intake (g.d^− 1^). (**D**) Average relative daily Totum-448 intake, normalized per body weight (g.kg body weight^− 1^.d^− 1^) No statistical analysis was carried out for Vex measurement. *N* = 12 animals. The average daily Totum-448 intake was compared with a Mann-Whitney or Student t test between groups WD-T448 and WD-Vex-T448. ND: normal diet. WD: western diet. WD-T448: western diet + Totum-448. WD-Vex: western diet + voluntary exercise. WD-Vex-T448: western diet + voluntary exercise + Totum-448.

Animals from groups WD-Vex and WD-Vex-T448 had unlimited access to the exercise wheel with a 2-day resting period each week. Data from day 54 and 55 are missing (week 8) due to an oversight in initiating the recording software. Additionally, a software crash due to a suspicion of computer capacity overload resulted in inconsistent recorded data from day 69 to 77 (over week 10 and 11). These data were excluded. The access to the Vex wheel remained constant despite recording failure. Included data were averaged by week and daily distance covered as a function of speed category is presented in Fig. 4A. Daily averages ranged from 11 to 17 km. Whilst no statistical analysis was carried out, no differences are apparent between groups and no decrease in speed over time was observed. Overall daily time spent running in the different speed categories is presented in Fig. 4B. Overall, animals from groups WD-Vex and WD-Vex-T448 respectively ran 22 and 21 min/day in the ⩽12 m/min category, 33 and 33 min/day in the [12–23] m/min speed category, 83 and 83 min/day in the [23–35] m/min category, 177 and 191 min/day in the [35–46] m/min category, and 45 and 28 min/day in the > 46 m/min speed category. While access to the wheel was granted 24 h per day, animals preferentially ran during the night phase (data not shown). Average Totum-448 intake over intervention period is presented in Fig. 4C. In line with the observed increase in food intake in exercising groups, the product intake was substantially higher in WD-Vex-T448 compared to WD-T448 (0.30 ± 0.03 vs. 0.23 ± 0.02 g/day, Fig. 4C), representing a 34% increase, or, if body-weight normalized, 2.46 ± 0.22 vs. 1.89 ± 0.18 g/day, Fig. 4D, representing a 17% increase.

### Indirect calorimetry and spontaneous locomotor activity

**Fig. 5:**
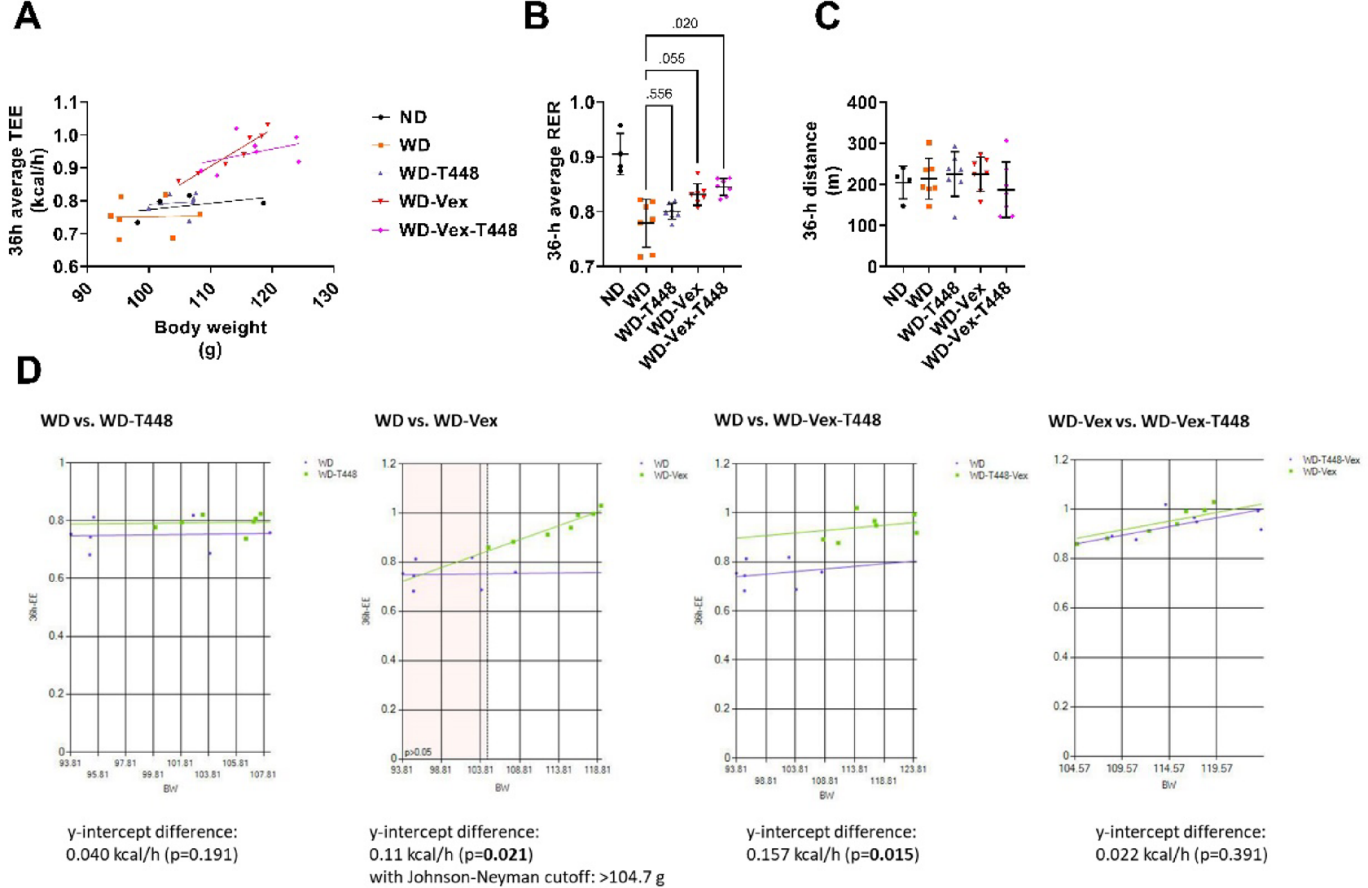
Analysis of TEE, RER and locomotor activity in calorimetric cages. Animals were put in calorimetric cages for 48 h between day 67 and 77. Gas exchanges and locomotor activity were assessed for 36 continuous hours (12 h-nighttime, 12 h-daytime, 12 h-nightime). Values of TEE, RER and locomotor activity are presented without any normalization^44^. A: 36-h average TEE as a function of body weight. B: 36-h average RER. C: 36-h total locomotory activity in the calorimetric cages, in absence of any exercise wheel. D: Pairwise comparison of 36-h average TEE with ANCOVA-calculated common slope, using body weight as a covariant: WD vs. WD-T448, WD vs. WD-Vex (*ANCOVA assumptions were not met for pair-wise comparison of groups WD and WD-Vex, slopes significantly differed between groups, *p* = 0.038, making it impossible to apply a common slope. Thus, ANCOVA was run using the Johnson-Neyman procedure with a cut-off of < 104.7 for covariant^47^, WD vs. WD-Vex-T448, and WD-Vex vs. WD-Vex-T448. Detailed pairwise ANCOVA results are provided in Suppl. Table 4. *N* = 4–7 animals. Pair-wise comparisons (Šidák’s, Dunnett’s, or Dunn’s post-hoc tests) are indicated only if one-way ANOVA, Welch-corrected one-way ANOVA, or Kruskal-Wallis test was significant, respectively (*p* < 0.05). No pairwise comparison with group ND was carried out, this group is shown for reference only. ANCOVA was carried out using the MMPC Multiple Linear Regression Output tool (http://www.mmpc.org/shared/regression.aspx). ND: normal diet. RER: respiratory exchange ratio. TEE: total energy expenditure. WD: western diet. WD-T448: western diet + Totum-448. WD-Vex: western diet + voluntary exercise. WD-Vex-T448: western diet + voluntary exercise + Totum-448.

The average hourly TEE over 36 h as a function of body weight is presented in Fig. 5A. Pairwise comparisons of 36-h average TEE with ANCOVA-calculated common slope, using body weight as a covariant are presented in Fig. 5B. TEE was significantly higher in the WD-Vex group compared to WD (+ 0.110 ± 0.029 kcal/h, *p* = 0.021, Fig. 5D) and in WD-Vex-T448 versus WD (+ 0.157 ± 0.054 kcal/h, *p* = 0.015, Fig. 5D). No significant differences were observed between WD and WD-T448 or between WD-Vex and WD-Vex-T448 (*p* = 0.191 and *p* = 0.391, respectively).

Average RER over 36 h was significantly increased in WD-Vex-T448 vs. WD (0.84 ± 0.02 vs. 0.78 ± 0.04, *p* = 0.020, Fig. 5B) while a non-statistical difference was observed for WD-Vex vs. WD (0.83 ± 0.02 vs. 0.78 ± 0.04, *p* = 0.055, Fig. 5B). Finally, no difference was observed among groups in 36-h total locomotory activity in the cage (Fig. 5C). The 12-h analysis (averaged over the day/night cycle) of TEE, RER, and locomotory activity is available in Suppl. Figures 1 and 2. Taken together, these results reveal that TEE (measured in absence of Vex wheel) was increased in exercising groups (WD-Vex and WD-Vex-T448) independently of body weight and spontaneous locomotor activity. RER was also increased in these groups (although not significantly in group WD-Vex, *p* = 0.055). Totum-448 alone did not significantly affect TEE, RER, or locomotor activity.

### Serum and liver lipids

**Fig. 6:**
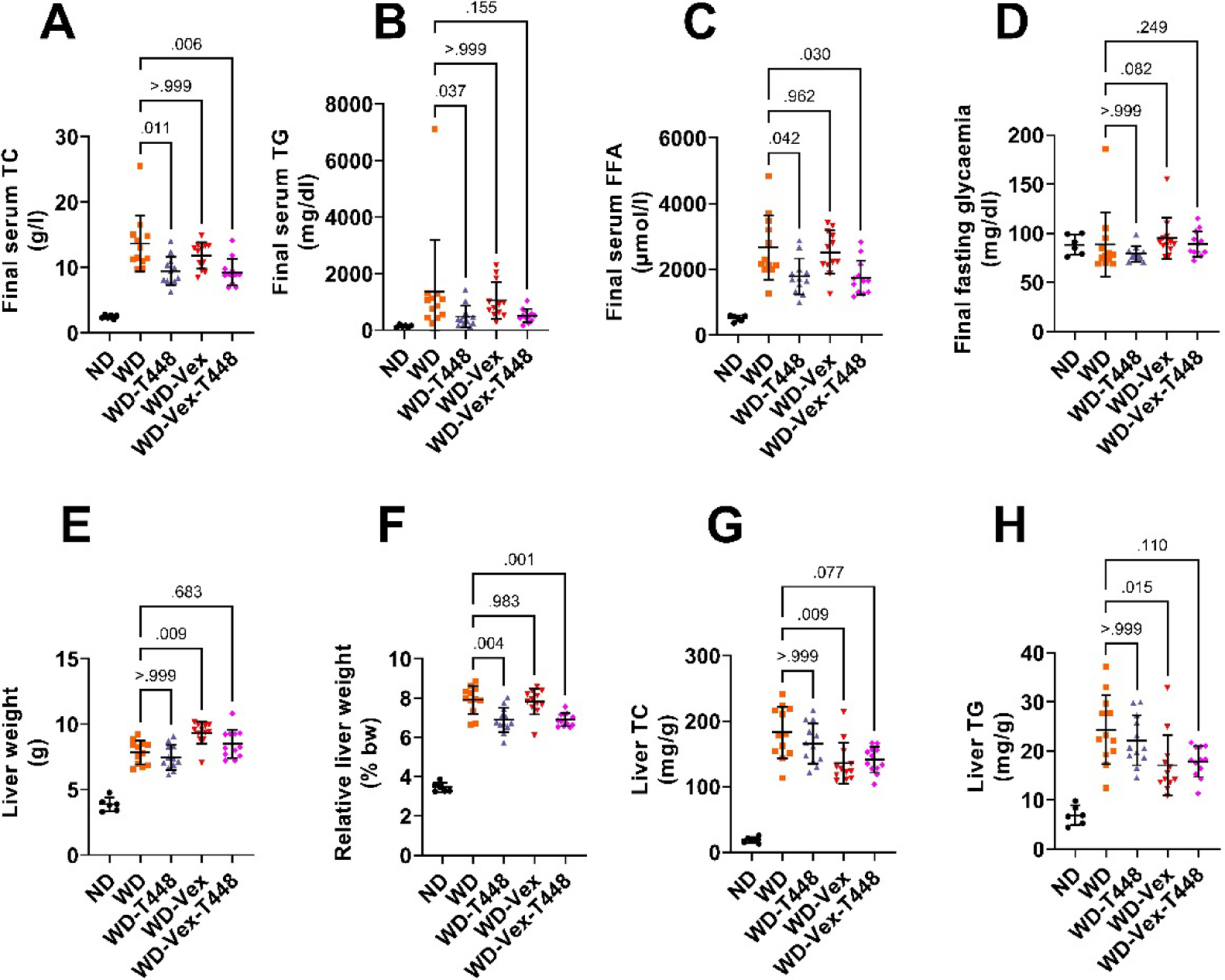
Serum and liver lipids following intervention, assessed at the end of the study (day 78 ± 1). (**A**) Final serum TC. (**B**) Final serum TG. (**C**) Final serum FFA. (**D**) Final fasting glycemia. (**E**) Liver weight. (**F**) Liver weight relative to body weight. (**G**) Liver TC, in mg per g of frozen tissue. (**H**) Liver TG, in mg/g of frozen tissue. *N* = 6–12 animals. Pair-wise comparisons (Šidák’s, Dunnett’s, or Dunn’s post-hoc tests) are indicated only if one-way ANOVA, Welch-corrected one-way ANOVA, or Kruskal-Wallis test was significant, respectively (*p* < 0.05). No pairwise comparison with group ND was carried out, this group is shown for reference only. FFA: Free fatty acids. ND: normal diet. TC: Total cholesterol. TG: Triglycerides. WD: western diet. WD-T448: western diet + Totum-448. WD-Vex: western diet + voluntary exercise. WD-Vex-T448: western diet + voluntary exercise + Totum-448.

At the end of the study, the WD-induced increase in serum TC was significantly blunted in both Totum-448 supplemented groups, WD-T448 and WD-Vex-T448, compared with WD (9.5 ± 2.2 and 9.3 ± 2.0 vs. 13.7 ± 4.3 g/l, *p* = 0.011 and *p* = 0.006, Cohen’s d: -1.22 and − 1.30, respectively, Fig. 6A). The increase in serum TG was significantly prevented only in the WD-T448 group, vs. WD (487.5 ± 377.6 vs. 1358.4 ± 1847.7 mg/dl, *p* = 0.037, Cohen’s d: -0.65, Fig. 6B). Similarly, the WD-induced rise in serum FFA was partially inhibited in both Totum-448 supplemented groups, WD-T448 and WD-Vex-T448, vs. WD (1786.9 ± 547.2 and 1742.5 ± 523.1 vs. 2666.0 ± 980.0 µmol/l, *p* = 0.042 and *p* = 0.030, respectively, Cohen’s d: -1.11 and − 1.18, Fig. 6C). As observed during the induction period, fasting glycemia was not significantly altered by WD exposure or by any intervention (Fig. 6D). Absolute liver weight was increased in WD-Vex, compared to WD (9.4 ± 0.8 vs. 7.8 ± 0.9 g, *p* = 0.009, Fig. 6E). However, when normalized to body weight, relative liver weight did not differ between these groups (7.8 ± 0.7 vs. 7.9 ± 0.7%, *p* = 0.983, Fig. 6F). In contrast, relative liver weight was significantly decreased in both WD-T448 and WD-Vex-T448, compared to WD (6.9 ± 0.6 and 6.9 ± 0.3 vs. 7.9 ± 0.7%, *p* = 0.004 and *p* = 0.001, respectively, Fig. 6F). Hepatic TC content was decreased in both exercising groups (WD-Vex and WD-Vex-T448) compared with WD, reaching statistical significance in WD-Vex only (135.9 ± 31.1 and 141.0 ± 19.7 vs. 182.8 ± 39.3 mg/g tissue *p* = 0.009 and *p* = 0.077, Cohen’s d: -1.32 and − 1.34, respectively, Fig. 6G). Likewise, hepatic TG content was significantly reduced in WD-Vex vs. WD (17.1 ± 6.2 vs. 24.3 ± 7.0 mg/ g tissue, *p* = 0.015, Cohen’s d: -1.09, Fig. 6H), while the difference between WD-Vex-T448 and WD did not reach statistical significance (17.9 ± 3.1 vs. 24.3 ± 7.0 mg/g tissue, *p* = 0.110, Cohen’s d: -1.20, Fig. 6H). Totum-448 supplementation alone did not significantly affect hepatic TC or TG levels. In summary, Totum-448 primarily exerted lipid-lowering effects on circulating parameters (TC, TG, and FFA), whereas voluntary exercise did not significantly affect serum lipids. Conversely, voluntary exercise improved hepatic lipid content (TC and TG), while Totum-448 had no detectable effect on liver lipid accumulation.

### Histological measurements and gene markers of inflammation, fibrosis, and oxidative stress

**Fig. 7:**
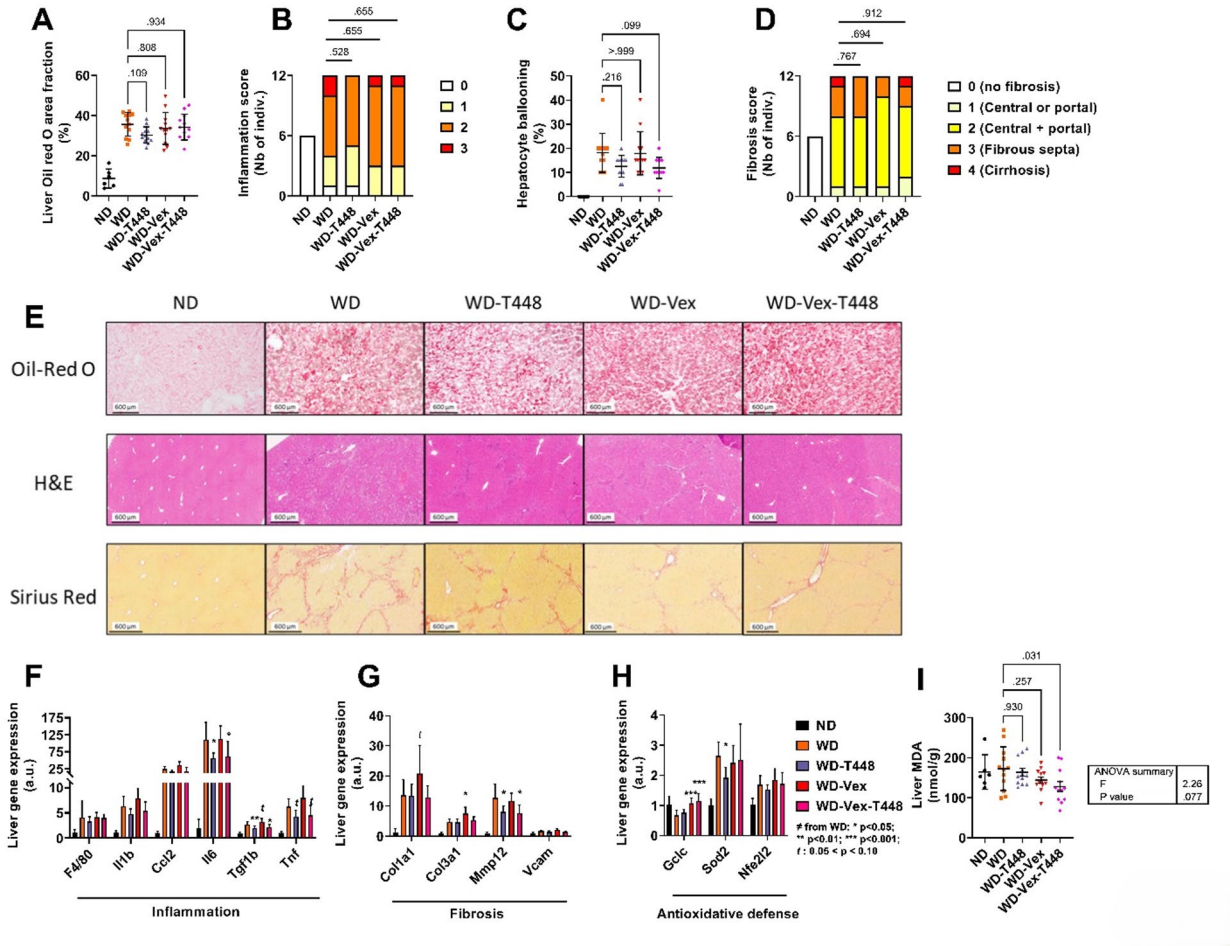
Hepatic histological measurements and gene markers of inflammation, fibrosis, and oxidative stress assessed at the end of the study (day 78 ± 1). (**A**) Liver Oil red O-stained area fraction (average of at least 3 images by sample). (**B**) Inflammation scoring assessed in H&E-stained liver sections in blinded condition, graded from 0 (no inflammation) to 3 (significant inflammation). (**C**) Percentage of ballooned hepatocytes assessed in blinded condition in H&E liver sections. (**D**) Fibrosis scoring of liver parenchyma, assessed in blinded condition in Sirius red-dyed sections. Fibrosis score corresponds to: 0 = none, 1 = mild (portal or pericellular fibrosis), 2 = moderate (thin and diffuse or thick but occasional fibrosis), 3 = thick and diffuse fibrosis, and 4 = cirrhosis. (**E**) Representative images of liver Oil red O, H&E, and Sirius Red-stained sections. Relative expression of genes associated with liver inflammation (**F**), fibrosis (**G**), and antioxidative defense (**H**). (**I**) Liver MDA levels, in nmol per g of frozen tissue. *N* = 6–12 animals. Pair-wise comparisons of continuous variables (Šidák’s, Dunnett’s, or Dunn’s post-hoc tests) are indicated only if one-way ANOVA, Welch-corrected one-way ANOVA, or Kruskal-Wallis test was significant, respectively (*p* < 0.05). Comparisons (p values) vs. group WD in graphs (**E**,**F**,**G**) are presented as: *t*: 0.05 < *p* < 0.10, *: *p* < 0.05, ***: *p* < 0.001. For categorical variables (**A**,**C**), pairwise Chi-square test values are indicated on the graph. No pairwise comparison with group ND was carried out, this group is shown for reference only. A.U.: Arbitrary units. MDA: Malondialdehyde. ND: normal diet. WD: western diet. WD-T448: western diet + Totum-448. WD-Vex: western diet + voluntary exercise. WD-Vex-T448: western diet + voluntary exercise + Totum-448.

Liver steatosis was assessed histologically by quantification of Oil red O-stained area fraction (Fig. 7A). While an increase in group WD was visible, no significant effect of any intervention, alone or in combination, was found. Inflammation and ballooning were assessed in histological H&E-stained liver sections. No improvement of inflammation was visible following Totum-448 or Vex (or their combination, Fig. 7B). A non-significant reduction of hepatocyte ballooning was observed in WD-Vex-T448, compared to WD (11.9 ± 4.4 vs. 18.1 ± 8.1%, *p* = 0.099, Fig. 7C). Fibrosis assessment using Sirius Red–stained liver sections revealed no beneficial effects of any intervention on fibrosis score (Fig. 7D). Representative Oil-Red O, H&E-, and Sirius Red–stained liver sections are shown in Fig. 7E. At the molecular level, the expression of genes associated with inflammation and fibrosis was elevated in WD animals, consistent with MASLD development. Despite the absence of histological improvement, Totum-448 supplementation in both WD-T448 and WD-Vex-T448 groups significantly reduced the relative expression of *Il6* (*p* = 0.021 and *p* = 0.034, respectively, Fig. 7F), *Tgf1b* (*p* = 0.005 and *p* = 0.027, respectively, Fig. 7F) and *Mmp12* (*p* = 0.018 and *p* = 0.012, respectively, Fig. 7G), while the reduction of *Tnf* relative expression was not significant (*p* = 0.080 and *p* = 0.098, Fig. 7F). Vex, on the other hand, did not induce any significant improvement in inflammatory or fibrotic gene expression. On the contrary, *Col3a1* expression was significantly increased in WD-Vex compared with WD (*p* = 0.013, Fig. 7G), while the increase in *Col1a1* was non-significant (*p* = 0.082 Fig. 7G). Additionally, we assessed the expression of genes associated with antioxidant defense. Relative expression of *Gclc*, encoding glutamate–cysteine ligase, a key enzyme in glutathione synthesis, was significantly increased in WD-Vex and WD-Vex-T448 compared with WD (*p* < 0.001 and *p* < 0.001, respectively, Fig. 7H). In contrast, *Sod2* expression, encoding mitochondrial superoxide dismutase, was significantly reduced in WD-T448 compared with WD (*p* = 0.011, Fig. 7H). Finally, hepatic MDA levels, a marker of lipid peroxidation, were significantly lower in WD-Vex-T448 compared with WD (128.3 ± 41.9 vs. 172.9 ± 53.6 nmol/g tissue, *p* = 0.031, Fig. 7I), although no increase associated with WD exposure was observed. However, this finding should be interpreted with caution, as the overall ANOVA did not reach statistical significance (*p* = 0.077), despite this significant pairwise post hoc comparison. In summary, Totum-448 modestly prevented WD-induced increases in the expression of certain inflammatory (*Il6*,* Tgf1b*) and fibrotic (*Mmp12*) gene markers, without detectable histological improvement, whereas voluntary exercise alone did not confer anti-inflammatory or antifibrotic benefits in this model.

## Discussion

We hypothesized in this work that only the combined intervention of Vex and Totum-448 would produce measurable benefits within this short timeframe, through additive improvements in hepatic (steatosis, inflammation, and fibrosis) and circulating (lipids) MASLD-related parameters. This hypothesis was not supported by our data. Instead, our results demonstrate distinct, intervention-specific effects of Totum-448 and Vex, without evidence of additive benefits when combined. Totum-448 primarily improved circulating lipid profiles, significantly reducing serum TC, TG, and FFA, and downregulated the expression of selected hepatic inflammatory and fibrotic gene markers. In contrast, Vex predominantly exerted beneficial effects on liver lipid content, decreasing hepatic TG and TC, while also improving body composition and increasing TEE. The observed standardized effect sizes for these significant findings ranged from 0.65 to 2.73, indicating robust biological effects. The combined intervention did not confer additive benefits beyond those observed with either intervention alone. However, when either intervention alone produced beneficial effects, these were generally preserved in the combined group, although statistical significance was not reached for certain outcomes, such as hepatic TG (*p* = 0.110) and TC (*p* = 0.077). Importantly, while the combination of Totum-448 and Vex clearly did not induce any additive effect, the proximity of these p-values to conventional significance thresholds suggests that a complete loss of effect in the combined intervention is unlikely and should be interpreted with caution.

Compared to previous studies investigating Totum-448 in the same model^8^, the present trial failed to replicate certain benefits of Totum-448 alone on MASLD features. Notably, liver steatosis was not reversed by the supplementation, in contrast to prior findings. This discrepancy may be explained by the shorter duration of supplementation in the current study (5 weeks versus 12 to 18 weeks previously) and by the experimental design, where MASLD was first induced by a WD for 6 weeks before initiating the intervention, making it a more severe pathological induction. Interestingly, and despite the absence of effects on liver steatosis, Totum-448 elicited modest improvements in liver inflammation and fibrosis at the gene level. Like in previous studies, this effect on gene markers did not translate into histologically visible changes^8^, only a non-significant reduction of ballooning (*p* = 0.099) was observed in the combined group. However, the modest histological changes indicate that this model primarily reflects an early, steatosis-dominant stage of MASLD rather than advanced MASH, which limits its ability to capture later fibrotic and inflammatory features of the disease and should be considered when interpreting the translational relevance of the finding.

Inflammatory pathways can in turn activate hepatic stellate cells and trigger the fibrotic response, via collagen-encoding genes^19^. The reduction of hepatic steatosis is often a primary driver for the subsequent attenuation of inflammation in MASLD. However, in this study, the observed modulation of inflammatory markers independently of any effect on steatosis may suggest a more direct anti-inflammatory action of certain bioactive compounds present in Totum-448. Numerous polyphenols are well documented for their anti-inflammatory properties through various mechanisms, including the modulation of the arachidonic acid pathway, the inhibition of excessive nitric oxide production, the regulation of key intracellular signaling cascades such as NF-κB and MAPK or the shift the balance between pro- and anti-inflammatory cytokine production^20^. While the present study was not designed to investigate these mechanisms in detail, several polyphenols included in Totum-448 have demonstrated such effects in previous research. Notably, oleuropein and its derivatives have been shown to modulate cytokine production in both in vitro and in vivo models^21^. Chlorogenic acid and anthocyanins have been linked to the inhibition of NF-κB pathway activation^22,23^ and luteolin has demonstrated inhibitory effects on both arachidonic acid metabolites and NF-κB signaling in vivo^24^. In addition, the anti-inflammatory potential of polyphenols may also be mediated, at least in part, through their antioxidant properties^25^. In this context, we observed a reduction in the relative expression of *Sod2*, which encodes the mitochondrial antioxidant enzyme superoxide dismutase 2. This enzyme plays a critical role in neutralizing superoxide anions by catalyzing their dismutation into molecular oxygen and hydrogen peroxide, thereby mitigating oxidative damage^26^. The upregulation of *Sod2* is a well-known cellular response to oxidative stress^27^, hence the elevated expression observed in the WD group compared to ND likely reflects a compensatory defense mechanism. In this framework, the reduced *Sod2* expression in the Totum-448 group could suggest a lower oxidative burden. Here again, the antioxidant properties of polyphenols have been extensively described and result from through scavenging of free radicals or inhibition of oxidative enzymes^25,28^. However, this interpretation must be tempered by the fact that no significant reduction in MDA (the only marker of oxidative damage measured in this work) was observed with Totum-448 alone.

Regarding Vex quantification, hamsters ran an average of 12–16 km per day, predominantly at speeds ranging from 34 to 46 m/min. This intensity was sustained for 177 and 191 min per day on average in the WD-Vex and WD-Vex+T448 groups, respectively, resulting in a cumulative daily running time reached approximately 6 h, with no apparent decline over the intervention period. Notably, Totum-448 supplementation did not impair exercise behavior or endurance. Published data on running speed and intensity in hamster voluntary wheel exercise remain scarce. Nevertheless, our findings are consistent with early studies in female golden Syrian hamsters, reporting daily running distances of up to 18 km, performed over 4 to 10 h at slightly lower average speeds (35–51 cm/s, corresponding to 21–31 m/min)^29^. It is worth noting, however, that methodological differences may complicate direct comparisons. In particular, the speed data in our study were derived using a 15-second bin size (a methodology that our team already published in a previous paper, in Sprague-Dawley rats^18^), which can influence how bursts of activity are averaged or detected. Longer bins may smooth out fluctuations, potentially underestimating peak intensities. It is not clear what time interval was used for speed measurement in the study by Borer et al. By comparison, forced treadmill exercise protocols in hamsters generally operate at lower intensities, with peak running speeds of 20–25 m/min sustained for about 1 h, typically on flat or mildly inclined (5°) surfaces^30,31,32^.

Functionally, Vex was associated with increased lean and muscle mass, along with a reduction in fat mass, particularly in perirenal adipose tissue. These changes likely reflect increased energy expenditure induced by voluntary activity, as well as elevated total energy expenditure even in animals without wheel access. While Totum-448 did not interfere with the increase in muscle mass and energy expenditure, the reduction of absolute fat mass and subsequent perirenal AT pad weight consecutive to Vex was hindered by the supplementation. While conflicting, these results should be interpreted in the context of the specific characteristics of this hamster model under WD feeding. Unlike other rodent models, WD exposure in Golden Syrian hamsters in this trial has led to only modest changes in overall fat mass and adipose tissue pad weights compared to animals on a ND. In contrast, WD-fed animals developed striking hepatic TG accumulation and markedly elevated circulating FFA. This metabolic profile could suggest a lipolytic state in adipose tissue^33,34^, potentially reflecting an impaired capacity for these animals to store excess lipids in fat fads. As a result, unbuffered lipids may accumulate ectopically in non-adipose organs such as the liver, contributing to the development and progression of MASLD. This pattern may therefore be specific to this model and does not necessarily mirror the pathophysiology observed in other preclinical models or in humans, warranting further comparative investigation. In line with this, and consistent with previous findings in the same model^8^, fasting glucose levels remained unchanged. Importantly, in this pilot study using this hamster model, we reported unaltered insulin levels despite the development of hepatic steatosis and inflammation. Therefore, in the present study, insulin levels were not assessed again, as existing evidence indicates that this model does not robustly recapitulate the insulin resistance component of the disease.

The lack of effect of Vex on several parameters, such as liver inflammation, fibrosis, and circulating lipids, was somewhat unexpected. However, this observation must be interpreted in light of the increased caloric intake observed in the exercising groups, which was on average 41–47% higher. In this hamster model, the WD is the primary (and in fact the sole) driver of MASLD development. As such, the elevated WD intake may have counteracted some of the expected metabolic benefits of exercise, thereby limiting its impact on hepatic and systemic outcomes, as observed before by our team in a different model^18^.

Several limitations of this work must be acknowledged. First, the novelty of the findings is relatively modest, as the effects of Totum-448 in hamster models have been previously demonstrated, and physical activity is a well-established strategy for the prevention and management of metabolic diseases, including MASLD. In this regard, the daily voluntary running distances covered by the animals were unexpectedly high, reaching approximately 10–15 km per day, corresponding to more than 350 min of activity per day. This level of physical activity substantially exceeds what is typically achievable or recommended in humans and therefore raises questions regarding the direct translatability of these findings (as mentioned before, current clinical guidelines for MASLD management generally recommend 150–200 min of moderate-intensity physical activity per week^10,11,12^). In addition, the Totum-448 mixture used in this study (5% w/w) led to an average dose of 225 mg.d^− 1^ and 300 mg.d^− 1^ in groups WD-T448 and WD-Vex-T448, respectively, representing a dose of 2.1 and 2.4 g.kg^− 1^.d^− 1^, for hamsters weighing in average 108 and 123 g (respectively) at the end of the study. The effects of Totum-448 are being assessed in humans in 2 clinical trials (published NCT06047847^35^, and on-going NCT06704321) using a dose of 4.3 g.d^− 1^, that is, ~ 0.07 g.kg^− 1^ for a 65-kg adult. Applying the interspecies dose conversion factor of 7.4 for hamsters^36^, this would correspond to an equivalent dose of ~ 0.5 g.kg^− 1^ in hamsters. Therefore, in comparison, the doses used in the present study are more than 4 times higher than the anticipated human equivalent dose. Although such cross‐species dose conversions should be interpreted cautiously, the preclinical results reported here will require confirmation in clinical settings using the intended human doses. Moreover, as discussed above, food intake was higher in exercising animals, resulting in a 34% increase in Totum-448 intake in the WD-Vex-T448 group, or a 17% increase when normalized to body weight. This difference may represent an important confounding factor when comparing these two groups. In this context, we cannot exclude the possibility that the reduction in hepatic MDA levels observed exclusively in the WD-Vex-T448 group was influenced, at least in part, by the higher intake of Totum-448, given its high polyphenol content and the well-documented antioxidant properties of several of its constituents (e.g.., oleuropein or chlorogenic acid^37,38^). Another limitation of this study is the limited mechanistic insight into the pathways through which Totum-448 exerts its biological effects. Totum-448 is a complex, plant-derived formulation, variability in its phytochemical composition between batches or across studies may affect reproducibility and could partly contribute to discrepancies with previously reported findings. Although the formulation is chemically characterized, such variability remains an inherent challenge when working with multi-component botanical extracts. Future studies focusing on the identification and evaluation of individual bioactive constituents, such as specific polyphenols, would help to strengthen mechanistic understanding and improve reproducibility.

To conclude, this study evaluated the effects of 5 weeks of Totum-448 supplementation, Vex, or their combination in a hamster model of diet-induced MASLD. To our knowledge, this is the first study to investigate the impact of voluntary exercise on MASLD-related features in hamsters. In addition, the effects of Totum-448 were assessed over a shorter intervention period (5 weeks) and following prior MASLD induction (6 weeks), conditions that had not been previously explored in this species. As developed hereabove, our main findings highlight distinct effects of Vex and Totum-448 on body composition, energy expenditure, circulating lipid profile, liver lipid content, hepatic inflammatory and fibrotic gene markers. Neither intervention produced measurable improvements in histological markers of inflammation or fibrosis, nor were additive effects observed when Vex and Totum-448 were combined. Despite the overall modest magnitude of the observed improvements, these findings support the notion that the combination of Totum-448 and Vex was the most effective strategy to elicit improvements on MASLD features. This superiority did not stem from additive or synergistic effects, as initially hypothesized, but rather from the conservation of benefits conferred by each intervention alone. Importantly, these results echo current recommendations from major health authorities, which emphasize the integration of physical activity with dietary interventions as the cornerstone of metabolic disease management. In this context, combining plant-based nutritional strategies such as Totum-448 with regular physical activity may represent a relevant and sustainable approach to MASLD prevention and care in humans.

## Methods

### Animals

All the animal procedures were approved by the local ethics committee (C2E2A, Auvergne, France, under the number #32212-2021063014575266 accepted on October 22nd, 2021), and comply with ARRIVE guidelines, with the IUCN Policy Statement on Research Involving Species at Risk of Extinction, the Convention on the Trade in Endangered Species of Wild Fauna and Flora, as well as the EU Directive 2010/63 for the protection of animals used for scientific purposes. Fifty-four 6-week-old male golden Syrian hamsters were provided by Janvier Labs (Le Genest-Saint-Isle, France). All the hamsters were individually housed at 22 °C under a standard 12 h light–12 h dark cycle. Upon arrival, hamsters were acclimatized for 2 weeks and fed a purified normal diet (ND, Table 1). Animals had access to food and water *ad libitum*. The number of animals per group was determined based on previous studies in hamsters^8,39^. It was reduced to *n* = 6 in group ND to minimize the total number of animals used, in accordance with the 3Rs^40^, because comparisons to that group did not constitute an objective in this work. Animals were monitored daily for morbidity, mortality or adverse clinical signs.

**Table 1:** Composition of diets.

Macronutrient	ND (D16010603)	WD (D99122211)
	% kcal	% kcal
Protein	20	20
Carbohydrate	70	35
Fat	10	45
kcal/g	**3.85**	**4.66**
*Ingredient*	*g*	*g*
Casein	200.00	200.00
L-Cystine	3.00	3.00
Corn starch	452.20	72.80
Maltodextrin 10	75.00	100.00
Fructose	0.00	172.80
Sucrose	172.80	0.00
Cellulose	50.00	50.00
Soybean oil	25.00	25.00
Coconut oil, hydrogenated	20.00	177.50
Mineral mix S10026	10.00	10.00
Dicalcium phosphate	13.00	13.00
Calcium carbonate	5.50	5.50
Potassium citrate	16.50	16.50
Vitamin mix V10001	10.00	10.00
Choline bitartrate	2.00	2.00
Cholesterol	0.00	12.50
Dye	0.00	0.05
Totum-448	0.00	0.00
Total	1055.00	870.65

### Experimental groups

After habituation, animals were randomly assigned to group ND (*N* = 6) or WD (Western-diet, *n* = 48) for 42 days to induce MASLD, based on body weight and fat mass using Randomice software (v.1.17)^41^. Following induction (on day 43), the animals from subgroup WD (*n* = 48) were randomly divided into 4 groups based on body weight and fat mass using the same procedure. The experimental groups were as follows: WD (*n* = 12), WD-T448 (WD supplemented with Totum-448 5% w/w, *n* = 12), WD-Vex (WD with access to a voluntary exercise wheel, *n* = 12), WD-Vex-T448 (WD supplemented with Totum-448, 5% w/w and access to a voluntary exercise wheel, *n* = 12). This study phase lasted for an additional 36 ± 1 days (78 ± 1 days in total). Animals belonging to group ND carried on with the same diet al.l throughout the trial.

### Diets

The purified diets were manufactured by Research Diets (New Brunswick, NJ, USA, their composition is available in Table 1). The relative amounts of essential nutrients (cellulose, vitamins, …) in both diets were matched by their total calorie content, not by weight^42^^42^. Totum-448 was provided by Valbiotis (Perigny, France) and incorporated into the WD at 5% w/w by Research Diets. Its chemical characterization is available in Suppl. Table 1.

### Voluntary exercise

After induction (from day 44 study until day 78 ± 1), the hamsters belonging to groups WD-Vex and WD-Vex-T488 had free access to a large scurry wheel (80859LS, Lafayette Instrument, Lafayette, IN, USA) connected to an electronic sensor and a computer. The exercise wheel was blocked for 2 days every 5 to 7 days to allow the animals to rest. The outside of the wheel was covered with a protection made from an opened 18-inch bike tire inner tube to prevent animals from injuries due to their legs getting stuck in the gaps between the rods. The number of wheel revolutions per 15 s was recorded constantly for the whole duration of the study with a dedicated software (Scurry Activity System, Lafayette Instrument, Lafayette, IN, USA) and data were averaged over 1 week. Running distance and speed were calculated every 15 s using wheel circumference (1.44 m). Speed data was then stratified into 5 intervals, as described previously^18^, readapted to match hamster speeds:

- [0–12] m/min, corresponding to [0–2] or (0 ≥ x ≥ 2) wheel revolutions per 15 s.
- [12–23] m/min, corresponding to [2–4] or (2 > x ≥ 4) wheel revolutions per 15 s.
- [23–35] m/min, corresponding to [4–6] or (4 > x ≥ 6) wheel revolutions per 15 s.
- [35–46] m/min, corresponding to [6–8] or (6 > x ≥ 8) wheel revolutions per 15 s.
- > 46 m/min, corresponding to all values strictly superior to 8 wheel revolutions per 15 s.

### Indirect calorimetry and locomotor activity

Due to scheduling constraints that limited the number of animals that could be included in this experiment, a subset of animals was selected for this analysis. A total of 32 animals (4/6 in group ND, 7/12 in all other groups were placed in calorimetric cages (Promethion 8-channel multiplex system provided by Sable Systems International, Las Vegas, NV, USA) for approximately 48 h between day 67 and day 77 for respiratory measurements and estimation of locomotor activity in absence voluntary wheel exercise. The selection of the animals for this analysis was made based on the exclusions of animals at the margins of the body weight distribution, within each group. Specifically, in the ND group, the animal with the highest and the one with lowest body weight were excluded, whereas in all other groups, the two animals with the highest and the three with the lowest body weights were excluded. Data from included and excluded animals are available in Suppl. Table 2. Animals were put in the calorimetric cage in the morning, typically around 9:00 and measurement started at the beginning of the first 12-h night cycle at 19:00, for 36 h (Two night cycles and one light cycle). Total energy expenditure (TEE) was calculated using Weir’s equation^43^ out of VO_2_ and VCO_2_ measurements for each cage sampled every 30 s. RER was calculated as the ratio of the volume of carbon dioxide (CO_2_) produced to the volume of oxygen (O_2_) used, or VCO_2_/VO_2_). Locomotor activity was estimated with an XYZ beam break array (Sable Systems, Las Vegas, NV, USA). A standard 12-h light/dark cycle was maintained throughout the calorimetry study. No further transformation of data was carried out. TEE is presented as average hourly energy expenditure over study period (kcal.h^− 1^) as a function of body weight (g) using ANCOVA^44^, (see statistics section).

### Body parameters and food intake

Body weight was recorded once weekly. Body composition (fat mass and lean mass) was determined by magnetic resonance imaging (Echo MRI, Zinsser Analytic, Fürstenfeldbruck, Germany). An additional body weight and composition assessment was made in animals just before entering the calorimetric cages. Food intake was measured thrice weekly.

### Glycemia, serum total cholesterol and triglycerides

Before (day − 1) and after induction (day 43), and before euthanasia (day 78 ± 1), blood was collected from the gum vein of 5-6-hour fasted animals and fasting glycemia was measured from a drop of fresh whole blood with a StatStrip Glucose Xpress (Nova Biomedical Corp., Waltham, CA, USA). The rest of the whole blood was left to clot at room temperature for 30 min before being centrifuged for 10 min at 2000 g. Serum (supernatant) was collected and stored at -80 °C until analysis. Serum total cholesterol (TC) levels were assessed using an enzymatic assay kit provided by Biolabo (#LP80106, Maizy, France). Serum samples were diluted 10 times in NaCl 0.9%, and absorbance was read in a multiplate reader (Spark 10 M, Tecan, Männedorf, Switzerland). Serum triglyceride (TG) levels were assessed using TG enzymatic assay kit provided by Cayman Chemical Company (#10010303, Ann Arbor, MI, USA). Serum samples were diluted 10 times in sodium phosphate assay buffer absorbance was read at 540 nm in a multiplate reader (Spark 10 M, Tecan, Männedorf, Switzerland). For all colorimetric measurements, samples whose technical duplicate did not fall within 20% of their coefficient of variation were removed.

### Euthanasia and tissue sampling

At the end of the study Animals were fasted for 5–6 h and anesthetized with isoflurane. Blood was collected by cardiac puncture and serum was prepared and stored as described hereabove. Anesthetized animals were euthanized by cervical dislocation and liver, soleus muscles, gastrocnemius muscle, inguinal, epididymal, and perirenal adipose tissue pads were sampled out, weighed and snap frozen into liquid nitrogen.

### Free fatty acids

Serum free fatty acids (FFA) were assessed directly in serum collected by cardiac puncture using a Free Fatty Acid quantification kit provided by Sigma-Aldrich (#MAK044, Merck, St Louis MO, USA) following the manufacturer instructions. Serum was assessed pure (group ND) or diluted 5 times into assay buffer (WD-exposed groups). Absorbance was read at 570 nm in a multiplate reader (Spark 10 M, Tecan, Männedorf, Switzerland). The samples whose technical duplicates did not fall within 20% of their coefficient of variation were removed.

### Liver lipids

Lipids were extracted from frozen livers (10 mg for WD-exposed groups, 100 mg for group ND) by homogenization in 1 mL of a solution containing 5% NP40 in water using a rotor homogenizer (Ultra-Turrax T10, IKA, Germany) for 20 s at maximum speed (30,000 rpm). Homogenization was visually assessed, and the process was repeated if any tissue fragments remained. Homogenates were heated to 80–100 °C for 2–5 min before being cooled to room temperature. The whole process (heating-cooling) was repeated once; then, samples were centrifugated (2000 g—2 min). Liver TG content was determined using an enzymatic assay kit provided by Cayman Chemical Company (#10010303, Ann Arbor, MI, USA) following the instructions, as described above. Liver TC level was assessed using an enzymatic assay kit provided by Biolabo (#LP80106, Maizy, France) following the manual, as described above. Liver FFA were assessed using a Free Fatty Acid quantification kit provided by Sigma‐Aldrich (#MAK044, Merck, St Louis MO, USA). For all colorimetric dosages in liver homogenate, samples whose technical duplicates did not fall within 20% of their coefficient of variation were removed.

### Liver malondialdehyde

Malondialdehyde (MDA) levels were assessed in frozen liver samples with a lipid peroxidation (MDA) assay kit provided by Abcam (ab118970, Abcam, UK) following the manufacturer’s instructions. Briefly, 10 mg of frozen tissue was homogenized in a glass potter in 300 µL of the provided lysis buffer in presence of Butylated hydroxytoluene, before being centrifuged at 13.000 g for 10 min. Absorbance was measured in the supernatant in a multiplate reader at 532 nm (Spark 10 M, Tecan, Männedorf, Switzerland). The samples whose technical duplicates did not fall within 20% of their coefficient of variation were removed.

### Gene expression in the liver

Total mRNA from the liver was extracted using TRIzol (Invitrogen, Life Technologies). cDNA was synthesized from 2 µg RNA with the High-Capacity cDNA transcription kit (Applied Biosystems, Life Technologies, CA, USA) in a T100 Thermal Cycler (Bio-Rad, Hercules, CA, USA) for 10 min at 25 °C, 120 min at 37 °C and 5 min at 85 °C, in the presence of a RNase inhibitor (Ambion 0.2U/µL, # AM2682, Thermo Fisher Scientific, MA USA). Real-time quantitative PCR amplification was carried out using the CFX system (Bio-Rad, USA) with SYBR Green probes (Eurofins, Ebersberg, Germany) and Sybr Green Master Mix (SYBR Select Master Mix, Thermo Fischer Scientific, MA, USA). The ΔΔCt method was used to quantify mRNA levels. Gene expression was normalized using *Hprt1* and as a housekeeping gene. Data are represented using the Rq which is normalized to the control group as: *Rq* = 2^−∆∆Ct^, where [ΔCt = Ct (target)-Ct (housekeeping gene); ΔΔCt = ΔCt (sample)‐ΔCt (control)]. The sequences of SYBR green probes used in this work are presented in Suppl. Table 3.

#### Histology

##### Oil Red O staining

Fresh liver samples were frozen in optimal cut temperature medium, and 10 μm tissue sections were cut with a cryostat and fixed in cold 10% formalin for 5 min. Slides were stained with Oil Red O at 60 °C in an oven for 8 min. Oil Red O areas were quantified using ImageJ (NIH, Bethesda, MD, USA). Stained areas of at least 10 μm² and with a circularity between 0.1 and 1 were selected for quantifying the Oil Red O surface as percentage of the total surface. At least three images per liver were analyzed.

##### Hematoxylin and Eosin (H&E) and Sirius red staining

Freshly dissected liver samples were fixed for 48 h at 4 °C in formalin (4% paraformaldehyde) and kept in ethanol 70% at 4 °C for at least 3 h (up to overnight) before automated tissue processing (HistoCore Pearl, Leica Biosystems, Nussloch, Germany). Sections were then embedded in paraffin and sections were cut at 4 μm with a microtome and stored before staining.

H&E staining:

Liver sections were heated at 56 °C for 15 min and dewaxed in xylene (3 baths: 10 min, then twice 5 min) then rehydrated in 4 successive ethanol baths (100%, 95%, 80%, and 70%) for 3 min each followed by 1 immersion in ultra-pure water (3 min). Thereafter, sections were stained in a bath composed of Gill II hematoxylin (#GHS232 Sigma-Aldrich, Merck, St Louis MO, USA) for 4 min, washed twice in water for 10 min each before being immersed in 0.25% eosin Y (#HT110116, Sigma-Aldrich, Merck, St Louis MO, USA) for 45 s. Finally, sections were dehydrated with ethanol (3 baths: 95%, 100%, and 100%, for 3 min each) and xylene (3 min), and slides were mounted with Eukitt medium (#03989 Sigma-Aldrich, Merck, St Louis MO, USA).

Sirius red dyeing:

Liver sections were dewaxed in xylene then rehydrated in 4 successive ethanol baths (100%, 95%, 80%, and 70%) for 10 s each. Thereafter, they were stained in Sirius red (#365548, Sigma-Aldrich, Merck, St Louis MO, USA) 0.1% in picric acid for 1 h, and, after removal of excess stain in acetic acid (0.5% in distilled water), sections were dehydrated with 4 ethanol baths (70%, 80%, 90%, and 100%) for 10 s followed by 2 xylene immersions (3 min each). Slides were then mounted with Eukitt medium.

Analysis:

Microtome blades were analyzed in blinded conditions for scoring of inflammation, ballooning and fibrosis, as described previously^8,45^. Inflammation was graded from 0 (no inflammation) to 3 (significant inflammation), fibrosis of the liver parenchyma was evaluated using a score corresponding to 0 = none, 1 = mild (portal or pericellular fibrosis), 2 = moderate (thin and diffuse or thick but occasional fibrosis), 3 = thick and diffuse fibrosis, and 4 = cirrhosis. The ballooning extent was assessed as the percentage of ballooned cells. In this hamster model, hepatic steatosis is predominantly microvesicular and is poorly detected by H&E staining. As a result, steatosis was consistently scored as 0 despite clear lipid accumulation evidenced by Oil Red O staining and biochemical lipid quantification; therefore, a NAS-like composite score including steatosis was not applied, and histological scoring was limited to inflammation, ballooning, and fibrosis.

### Statistics

Values are presented as the mean ± SEM. GraphPad Prism V9.50 (San Diego, CA, USA) was used to draw figures and to run statistic comparisons. The differences were considered statistically significant at *p* < 0.05. For comparisons of means between groups: a Shapiro-Wilk normality test was used to determine whether the data are consistent with a Gaussian distribution. If data were not distributed according to the normal distribution, a Mann-Whitney test (when comparing only two groups, after MASLD induction), or a Kruskal-Wallis nonparametric test was used followed by Dunn’s test for post hoc comparisons. When normal distribution was assumed, measures were subjected to an unpaired t-test or Welsh-corrected t-test (when comparing only two groups, after MASLD induction), or a one-way ANOVA or Welch-corrected one-way ANOVA (if conditions of equivalence of variances were not met), followed by Šídák’s or Dunnett’s test for multiple comparisons, respectively, when ANOVA reached significance threshold. Only the following pairwise post-hoc comparisons were carried out: WD vs. WD-T-448, WD vs. WD-Vex, and WD vs. WD-Vex-T-448. No comparison was made with group ND (except for MASLD induction measurements). For group comparisons of data collected over time, statistic tests were carried out only at the final timepoint or average, and comparison of groups was performed as described there-above. Comparison of categorical data (inflammation and fibrosis histological scores) were carried out with pairwise chi-squared tests. ANCOVA was used for comparison of TEE using body weight as a covariant. The EE ANCOVA analysis done for this work was provided by the NIDDK Mouse Metabolic Phenotyping Centers (MMPC, www.mmpc.org) using their Energy Expenditure Analysis page (http://www.mmpc.org/shared/regression.aspx) and supported by grants DK076169 and DK115255. For the main quantitative significant outcomes, a standardized effect size (Cohen’s d) is also presented^46^.

## Supplementary Information

Below is the link to the electronic supplementary material.

Supplementary Material 1

## Data Availability

The data that support the findings of this study are available from the corresponding author upon reasonable request.
