# Supplementary material for: Differential effects of voluntary exercise and Totum-448, a plant-based formulation, in a hamster model of MASLD
Source: Sci Rep. 2026 Mar 9;16:12813. doi: 10.1038/s41598-026-43177-5 (PMC13096555; doi:10.1038/s41598-026-43177-5)
Supplement: Supplementary file 1 — Supplementary Material 1 [file 41598_2026_43177_MOESM1_ESM.docx]

**Supplementary Table 1**: Chemical characterization of Totum-448

Suppl. Table 1 shows the chemical characterization of TOTUM-448. Total phenolic compound levels (in gallic acid equivalent) was assessed using the Folin-Ciocalteu colorimetric method (Singleton et Rossi 1965). A more precise characterization of phytochemical compounds was performed by HPLC-UV/Visible/RID-MS using 1260 LC system and 1200 LC system with a 6110 Single Quad MS-ESI detector (Agilent Technologies, Santa Clara, CA, USA) with a C18 Prodigy reversed-phase column (250 mm × 4.6 mm, 5 μm; Phenomenex, USA) and an Atlantis HILIC Silica column (150×4.6 mm, 5 μm, Waters, The Netherlands).

Singleton, V. L., et Joseph A. Rossi. 1965. « Colorimetry of Total Phenolics with Phosphomolybdic-Phosphotungstic Acid Reagents ». *American Journal of Enology and Viticulture* 16 (3): 144‑58. https://doi.org/10.5344/ajev.1965.16.3.144.

| **Compound types (sorted by families)** | **Extract content (g/100 g)** |
| --- | --- |
| Choline* | 13.67 |
| Total phenolic compounds | 8.7 |
| Total anthocyanins | 0.536 |
| Monocaffeoylquinic acids |  |
| Chlorogenic acid | 0.517 |
| Cryptochlorogenic acid | 0.324 |
| Neochlorogenic acid | 0.319 |
| Other monocaffeoylquinic acids | 0.115 |
| Dicaffeoylquinic acids |  |
| Cynarine | 0.229 |
| 4,5-Dicaffeoylquinic acid | 0.098 |
| 3,5-Dicaffeoylquinic acid | 0.074 |
| 3,4-Dicaffeoylquinic acid | 0.056 |
| Caffeic acid | 0.008 |
| Oleuropein | 6.223 |
| Oleuropein isomers | 0.757 |
| Ligstroside | 0.131 |
| Luteolin | 0.017 |
| Luteolin-7-O-glucoside | 0.880 |
| Luteolin-7-O-glucuronide | 0.277 |
| Luteolin-4-O-glucoside | 0.083 |
| Apigenin-7-O-glucoside | 0.062 |
| Apigenin-7-O-glucuronide | 0.139 |
| Apigenin-7-O-rutinoside | 0.037 |
| Verbascoside | 0.152 |
| Terpenes and terpenoids |  |
| Oleanolic acid | 0.199 |
| Cynaropicrin | 0.139 |
| Saponins |  |
| Chrysanthellin A | 0.133 |
| Chrysanthellin B | 0.215 |
| Alkaloids |  |
| Piperin | 0.044 |

* in choline chloride equivalent.

***Supplementary. Table 2:*** *Data used for inclusion of animals in the indirect calorimetric procedure.*

Due to schedule constraints, indirect calorimetry experiment could only be assessed in 32 (out of 54) animals (4 rounds of 8 calorimetric cages). We therefore included 4/6 animals in group ND and 7/12 in all other groups. We excluded from this measurement animals having extreme body weight values within each group, as follows:

• Group ND: exclusion of the animal with highest body weight and the one with lowest body weight.

• All other groups: exclusion of the 2 animals with highest body weight and the 3 animals with lowest body weight, within each group.

Animals excluded are highlighted in blue (lowest body weight) or orange (highest).

| ND | | | | | | | | |
| --- | --- | --- | --- | --- | --- | --- | --- | --- |
| # | BW | FAT | | LEAN | | | Included? | |
| 1 | 100.88 | 12.19 | | 80.06 | | | Yes | |
| 2 | 100.23 | 13.26 | | 80.18 | | | Yes | |
| 3 | 99.19 | 12.42 | | 80.4 | | | No | |
| 4 | 120.66 | 10.66 | | 104.35 | | | No | |
| 5 | 119.66 | 16.15 | | 99.53 | | | Yes | |
| 6 | 103.16 | 10.96 | | 85.35 | | | Yes | |
|  | | | | BW | | FAT | LEAN | |
| Group mean | | | | 107.30 | | 12.61 | 88.31 | |
| Included mean | | | | 105.98 | | 13.14 | 86.28 | |

| WD | | | | |
| --- | --- | --- | --- | --- |
| # | BW | FAT | LEAN | Included? |
| 7 | 93.57 | 13.98 | 73.885 | Yes |
| 8 | 99.60 | 12.92 | 80.81 | Yes |
| 9 | 106.47 | 15.62 | 84.21 | Yes |
| 10 | 101.35 | 18.16 | 77.62 | Yes |
| 11 | 108.38 | 20.19 | 80.76 | No |
| 12 | 94.45 | 16.69 | 68.12 | Yes |
| 13 | 94.64 | 14.62 | 75.29 | Yes |
| 14 | 113.22 | 18.44 | 87.87 | No |
| 15 | 93.08 | 14.61 | 73.1 | Yes |
| 16 | 88.51 | 10.38 | 73.57 | No |
| 17 | 87.64 | 15.27 | 66.22 | No |
| 18 | 91.94 | 10.19 | 76.25 | No |
|  | BW | FAT | LEAN |  |
| Group mean | 97.74 | 15.09 | 76.48 |  |
| Included mean | 97.59 | 15.23 | 76.15 |  |

| WD-T448 | | | | |
| --- | --- | --- | --- | --- |
| # | BW | FAT | LEAN | Included? |
| 19 | 103.76 | 15.03 | 83.69 | Yes |
| 20 | 108.59 | 16.8 | 87 | No |
| 21 | 99.58 | 17.71 | 76.54 | Yes |
| 22 | 120.81 | 18.52 | 93.24 | No |
| 23 | 106.19 | 17.01 | 80.99 | Yes |
| 24 | 101.24 | 16.83 | 79.77 | Yes |
| 25 | 99.74 | 18.08 | 76.67 | No |
| 26 | 104.63 | 16.91 | 82.19 | Yes |
| 27 | 92.52 | 16.68 | 71.8 | No |
| 28 | 105.41 | 16.89 | 84.44 | Yes |
| 29 | 103.84 | 15.91 | 83.38 | Yes |
| 30 | 99.08 | 13.77 | 79.75 | No |
|  | BW | FAT | LEAN |  |
| Group mean | 103.78 | 16.68 | 81.62 |  |
| Included mean | 103.52 | 16.61 | 81.57 |  |

| WD-Vex | | | | |
| --- | --- | --- | --- | --- |
| # | BW | FAT | LEAN | Included? |
| 31 | 98.95 | 6.94 | 86.08 | No |
| 32 | 96.34 | 7.73 | 80.52 | No |
| 33 | 106.33 | 11.63 | 86.31 | Yes |
| 34 | 101.50 | 7.92 | 87.07 | Yes |
| 35 | 99.20 | 7.51 | 87.71 | No |
| 36 | 109.76 | 16.99 | 87.39 | No |
| 37 | 109.01 | 7.665 | 94.115 | Yes |
| 38 | 106.25 | 10.39 | 91.08 | Yes |
| 39 | 109.68 | 11.72 | 90.83 | Yes |
| 40 | 109.73 | 7.05 | 95.59 | Yes |
| 41 | 103.29 | 7.07 | 92.09 | Yes |
| 42 | 108.47 | 1.51 | 104.33 | No |
|  | BW | FAT | LEAN |  |
| Group mean | 104.88 | 8.68 | 90.26 |  |
| Included mean | 106.54 | 9.06 | 91.01 |  |

| WD-Vex-T448 | | | | |
| --- | --- | --- | --- | --- |
| # | BW | FAT | LEAN | Included? |
| 43 | 107.95 | 7.28 | 95.7 | Yes |
| 44 | 128.87 | 14.78 | 108.5 | No |
| 45 | 115.72 | 12.1 | 98 | No |
| 46 | 118.11 | 7.43 | 105.25 | Yes |
| 47 | 107.46 | 10.69 | 91.21 | Yes |
| 48 | 98.31 | 8.6 | 82.37 | No |
| 49 | 97.35 | 11.46 | 80.38 | No |
| 50 | 104.61 | 5.08 | 100.45 | Yes |
| 51 | 109.80 | 7.9 | 97.51 | Yes |
| 52 | 90.09 | 8.67 | 77.36 | No |
| 53 | 107.01 | 7.74 | 94.82 | Yes |
| 54 | 99.01 | 9.98 | 85.56 | Yes |
|  | BW | FAT | LEAN |  |
| Group mean | 107.02 | 9.31 | 93.09 |  |
| Included mean | 107.37 | 8.68 | 94.75 |  |

***Supplementary Table 3:*** *Sybr Green probes sequences used in this study.*

| **Target gene** | **Forward sequence** | **Reverse sequence** |
| --- | --- | --- |
| *Hprt1* | TGCTGACCTGCTGGATTACA | TTGAGAGATCGTCTCCACCAAT |
| *Tnf* | CAACCCTATCATCGGCTCCA | GCTCCTCCACTTGGTGGTTT |
| *Ccl2* | TGCTAACTTGACGCAAGCTC | AAGTTCTTGAGTCTGCGGTGG |
| *Il1b* | GCAACTGTTCCTGAACTCGA | TGGATAGCTCAGGTCAAGGCT |
| *Il6* | CCTGGAGTTTGTGACGAACAAT | GTTGGGCTAGGCGTGACTATT |
| *F4/80* | GCTTCCAACCAGAGCCAGAA | CCTGCTTGGCACTGCTGTAT |
| *Gclc* | TGGGATTTGGGATGGGCAAT | GCAGCACTCAAAGCCATAACA |
| *Sod2* | CTACGTGAACAACCTGAACGCC | TTGGGCTCTCCACCACCATTAG |
| *Nfe2l2* | GATCCTCAGTGCAGCGTTCG | TCAAATCCATGTCCTGCTGGG |
| *Col1a1* | CTGACGCATGGCCAAGAAGA | CGTGCCATTGTGGCAAATACA |
| *Col3a1* | GGCTCTCCTGGAATCTGTACA | GGATAGCCACCAATTCCTCCT |
| *Mmp12* | TCCTGTTTGCATCTGGAGTTCA | GCTCGTGAACAGCAACAAGG |
| *Vcam* | ACTGCAAGTCTACACATCTCCC | GGTAGACCCTCACTGGAGCA |

***Supplementary Table 4:*** *Results of pairwise ANCOVA of 36-h TEE using body weight as a covariant.*

| **TEE averaged over 36h** | | | Covariant: BW |  |  |  |  |
| --- | --- | --- | --- | --- | --- | --- | --- |
| *Group A* | *Group B* | *Slope comparison (p)* | *y-intercept difference (kcal/h)* | *y-intercept comparison (p)* |  |  |  |
| WD | WD-Vex | 0.038* | 0.110 ± 0.029 | **0.021** | J-N cut-off >104.7 | | |
| WD | WD-Vex-T448 | 0.560 | 0.157 ± 0.054 | **0.015** |  |  |  |
| WD | WD-T448 | 0.910 | 0.040 ± 0.029 | 0.191 |  |  |  |
| WD-Vex | WD-Vex-T448 | 0.086 | 0.022 ± 0.024 | 0.391 |  |  |  |
|  |  |  |  |  |  |  |  |
|  |  |  |  |  |  |  |  |
| * ANCOVA assumptions not met (slopes were significantly different between groups p=0.038, making it impossible to apply a common slope) | | | | | | | |
| ANCOVA was run using the Johnson-Neyman procedure with a cut-off of >104.7 for the covariant. | | | | | | |  |

***Supplementary Figure 1:*** *TEE, averaged over 12 hours (by light/night cycle).*

Pairwise comparison of 12-h (daytime and nighttime) average TEE with ANCOVA-calculated common slope, using body weight as a covariant: WD vs. WD-T448, WD vs. WD-Vex (*ANCOVA assumptions were not met for pair-wise comparison of groups WD and WD-Vex in daytime, slopes significantly differed between groups, p=0.024, making it impossible to apply a common slope. Thus, ANCOVA was run using the Johnson-Neyman procedure with a cut-off of <104 for covariant [47]), WD vs. WD-Vex-T448, and WD-Vex vs. WD-Vex-T448. Detailed pairwise ANCOVA results are provided in Suppl. Table 5.


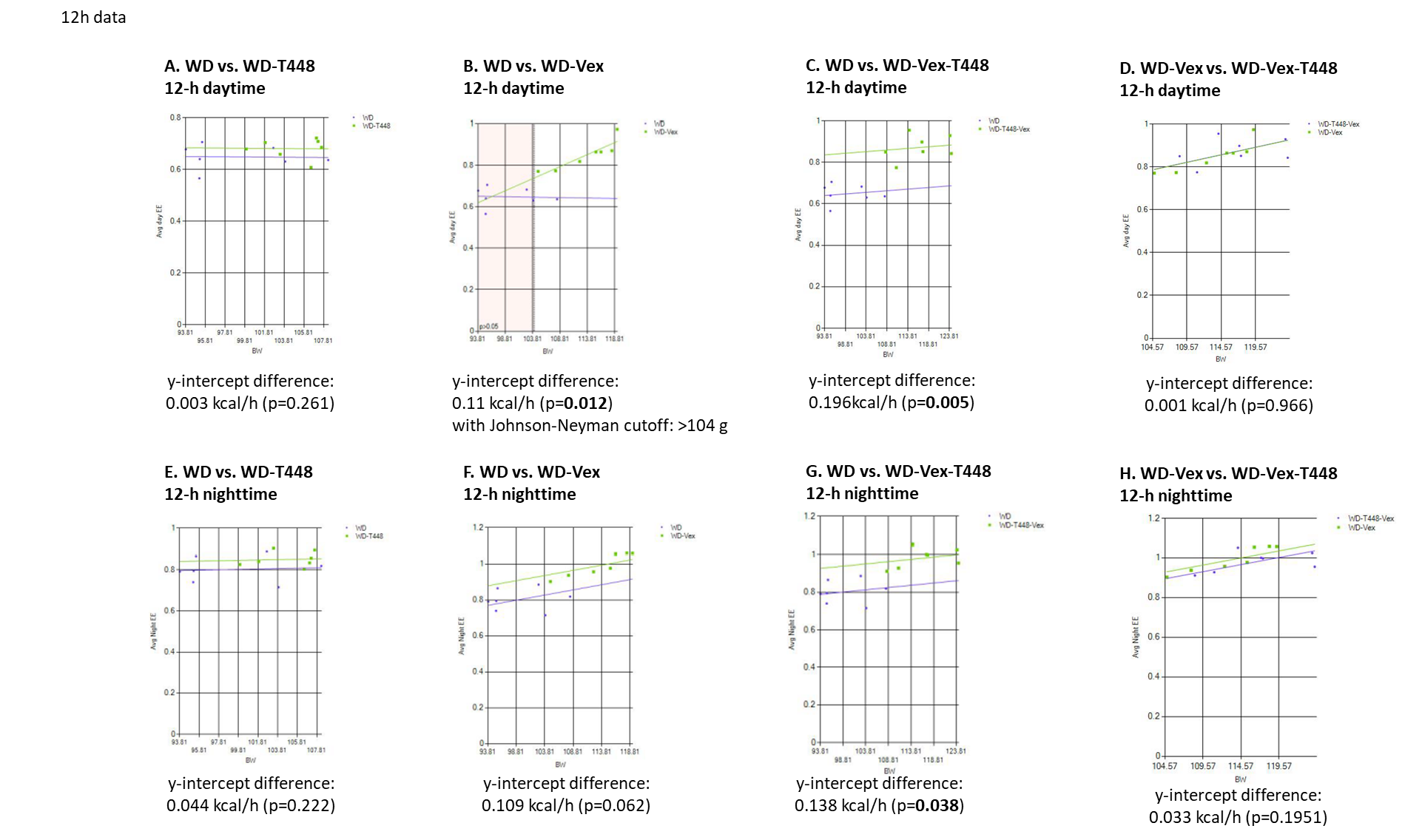


***Supplementary Figure 2:*** *RER and walking distance, averaged over 12 hours (by light/night cycle).*

Animals were put in calorimetric cages for 48h between day 67 and 77. Gas exchanges and locomotor activity were assessed for 36 continuous hours (12h-nighttime, 12h-daytime, 12h-nightime). Values of TEE, RER and locomotor activity are presented without any normalization. A: Average 12-h daytime RER. B: Average 12-h nighttime RER. C: 12-h daytime average total locomotory activity in the calorimetric cages, in absence of any exercise wheel. D: 12-h nighttime total locomotory activity in the calorimetric cages, in absence of any exercise wheel. N=4-7 animals. Pair-wise comparisons (Šidák’s, Dunnett’s, or Dunn’s post-hoc tests) are indicated only if one-way ANOVA, Welch-corrected one-way ANOVA, or Kruskal-Wallis test was significant, respectively (p<0.05). No pairwise comparison with group ND was carried out, this group is shown for reference only. ND: normal diet. RER: respiratory exchange ratio. WD: western diet. WD-T448: western diet + Totum-448. WD-Vex: western diet + voluntary exercise. WD-Vex-T448: western diet + voluntary exercise + Totum-448.

***Supplementary Table 5:*** *Results of pairwise ANCOVA of 12-h TEE (by light/night cycle), using body weight as a covariant.*

|  | |  |  |  |  |  |  |  |  |  |  |  |  |
| --- | --- | --- | --- | --- | --- | --- | --- | --- | --- | --- | --- | --- | --- |
| **TEE averaged over 12 hours (daytime)** | | | Covariant: BW | |  |  |  |  |  |  |  |  |  |
| *Group A* | *Group B* | *Slope comparison (p)* | *y-intercept difference (kcal/h)* | *y-intercept comparison (p)* |  |  |  |  |  |  |  |  |  |
| WD | WD-Vex | 0.024* | 0.12 ± 0.028 | **0.012** | Johnson-Neyman cut-off >104 | | |  |  |  |  |  |  |
| WD | WD-Vex-T448 | 0.526 | 0.196 ± 0.056 | **0.005** |  |  |  |  |  |  |  |  |  |
| WD | WD-T448 | 0.915 | 0.034 ± 0.283 | 0.261 |  |  |  |  |  |  |  |  |  |
| WD-Vex | WD-Vex-T448 | 0.137 | 0.001 ± 0.030 | 0.966 |  |  |  |  |  |  |  |  |  |
|  |  |  |  |  |  |  |  |  |  |  |  |  |  |
|  |  |  |  |  |  |  |  |  |  |  |  |  |  |
| * ANCOVA assumptions not met (slopes were signficantly different between groups, making it  impossible to apply a common slope) | | | | | | | | | | |  |  |  |
| ANCOVA was run using the Johnson-Neyman procedure with a cut-off of >104 for the covariant. | | | | | | | |  |  |  |  |  |  |
| \| **TEE averaged over 12 hours (nighttime)** \| \| \| Covariant: BW \| \| \| --- \| --- \| --- \| --- \| --- \| \| *Group A* \| *Group B* \| *Slope comparison (p)* \| *y-intercept difference (kcal/h)* \| *y-intercept comparison (p)* \| \| WD \| WD-Vex \| 0.079 \| 0.109 ± 0.052 \| 0.062 \| \| WD \| WD-Vex-T448 \| 0.606 \| 0.138 ± 0.058 \| **0.038** \| \| WD \| WD-T448 \| 0.920 \| 0.044 ± 0.034 \| 0.222 \| \| WD-Vex \| WD-Vex-T448 \| 0.100 \| 0.033 ± 0.024 \| 0.195 \| | | | | | | | | | | | | | |
